# Human-Centered Design Lessons for Implementation Science: Improving the Implementation of a Patient-Centered Care Intervention

**DOI:** 10.1097/QAI.0000000000002216

**Published:** 2019-11-26

**Authors:** Laura K. Beres, Sandra Simbeza, Charles B. Holmes, Chanda Mwamba, Njekwa Mukamba, Anjali Sharma, Virginia Munamunungu, Monica Mwachande, Kombatende Sikombe, Carolyn Bolton Moore, Aaloke Mody, Aybüke Koyuncu, Katerina Christopoulous, Lazarus Jere, Jake Pry, Peter D. Ehrenkranz, Ashwin Budden, Elvin Geng, Izukanji Sikazwe

**Affiliations:** a Department of Interna6onal Health, Johns Hopkins Bloomberg School of Public Health, Baltimore, MD;; b Centre for Infectious Disease Research in Zambia, Lusaka, Zambia;; c Georgetown University, Washington, DC;; d University of California San Francisco, San Francisco, CA;; e The Bill & Melinda Gates Foundation, Seattle, WA; and; f D'EVA Consulting, Washington, DC.

**Keywords:** HIV, human-centered design, implementation, Zambia

## Abstract

Evidence-based HIV interventions often fail to reach anticipated impact due to insufficient utilization in real-world health systems. Human-centered design (HCD) represents a novel approach in tailoring innovations to fit end-users, narrowing the gap between efficacious interventions and impact at scale.

**Methods:**

We combined a narrative literature review of HCD in HIV programs with our experience using HCD to redesign an intervention promoting patient-centered care (PCC) practices among health care workers (HCW) in Zambia. We summarize the use and results of HCD in the global HIV response and share case study insights to advance conceptualization of HCD applications.

**Results:**

The literature review identified 13 articles (representing 7 studies) on the use of HCD in HIV. All studies featured HCD hallmarks including empathy development, user-driven inquiry, ideation, and iterative refinement. HCD was applied to mHealth design, a management intervention and pre-exposure prophylaxis delivery. Our HCD application addressed a behavioral service delivery target: changing HCW patient-centered beliefs, attitudes, and practices. Through in-depth developer–user interaction, our HCD approach revealed specific HCW support for and resistance to PCC, suggesting intervention revisions to improve feasibility and acceptability and PCC considerations that could inform implementation in transferable settings.

**Conclusions:**

As both a research and implementation tool, HCD has potential to improve effective implementation of the HIV response, particularly for product development; new intervention introduction; and complex system interventions. Further research on HCD application strengths and limitations is needed. Those promoting PCC may improve implementation success by seeking out resonance and anticipating the challenges our HCD process identified.

## Abstract 2

Supplemental Digital Content is Available in the Text.

## INTRODUCTION

Although today's public health response to HIV has a robust set of evidence-based tools with which to address the global epidemic [eg, antiretroviral therapy (ART), pre-exposure prophylaxis (PrEP), and voluntary medical male circumcision], implementation has failed to achieve the tools' full preventive and therapeutic potential.^1^ Failures of implementation often result from inadequate fit between available innovations and the people, processes, and contexts in which they are delivered. Although an emergency-based response focusing on access largely drove HIV service delivery strategies over the past three decades, future success depends on more effectively engaging end-users with appropriate, desirable, and accessible services.^1,2^ For example, although ART is lifesaving, both treatment initiation and retention remain suboptimal, leading to onward transmission, morbidities, and mortality.^3^ These gaps demand alternative and innovative treatment delivery models—drawing from interdisciplinary perspectives—for advancing the public health response.^4,5^

Human-centered design (HCD) is an emerging approach with roots in industrial design, engineering, psychology, anthropology, business, computer science, ergonomics, and design that hold promise for improving implementation of evidence-based interventions for health. HCD brings end-users and developers together to cocreate health products, services, or delivery strategies that identify, prioritize, and address barriers to usability.^6–8^ Traditionally, HCD focused on product development using participatory activities emphasizing researcher and user interaction to improve intervention utility, uptake, sustainability, and effectiveness.^7–9^ Although no single definition of HCD in health exists,^6^ there are hallmarks present across HCD applications^7–9^ (Table 1). HCD uses methods likely familiar to social scientists working in health^8,10–13^ but emphasizes bringing the researchers and users together in a more empathetic way, generating breadth and flexibility in the investigation and prioritizes action over furthering scientific knowledge (Table 1, Tolley^8^).

**TABLE 1.**
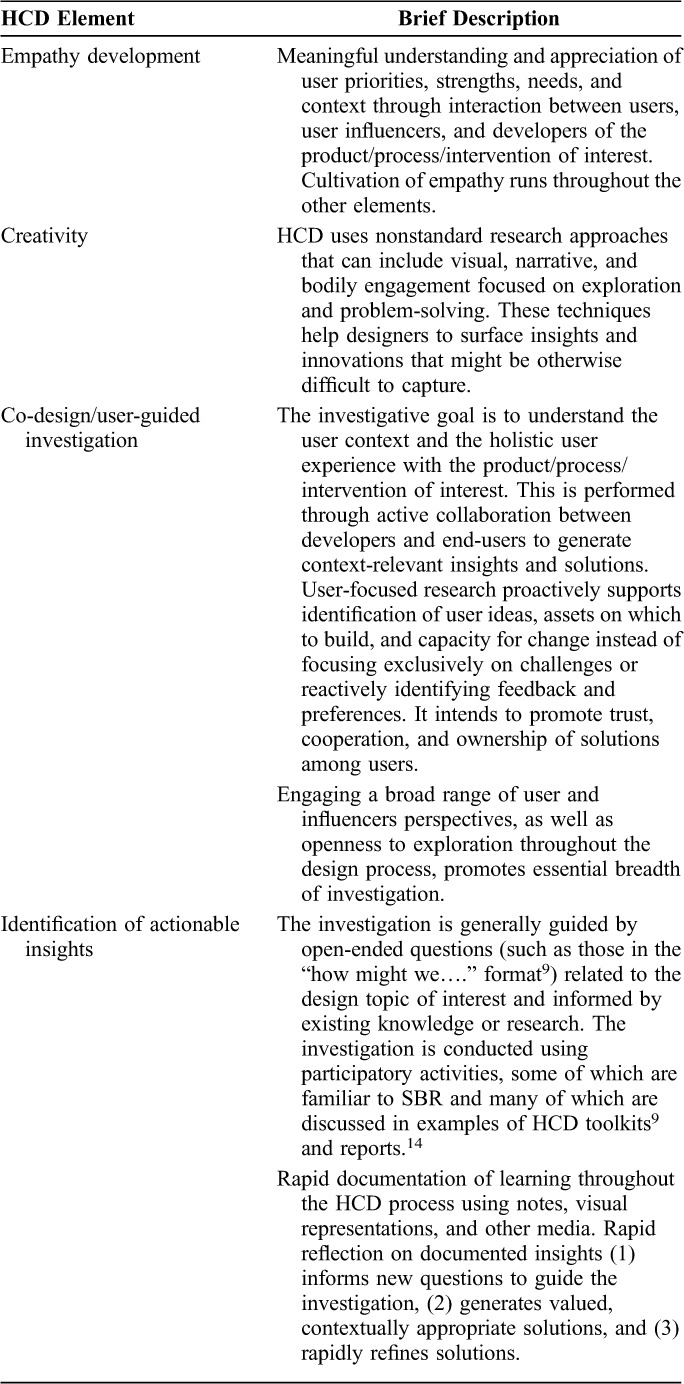

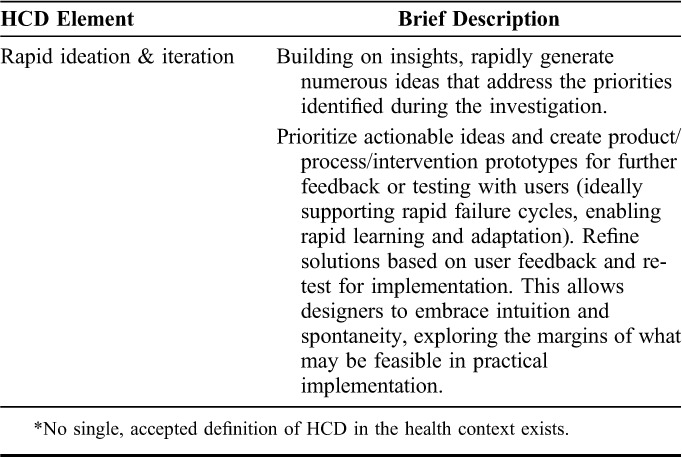
Summarized Key Elements of a HCD Process*

Despite a growing literature on HCD in health,^6^ no synthetic appraisal of HCD in the public health HIV response yet exists. In this article, we seek to advance the conceptualization of the use of HCD to address HIV through both a literature review and a case study of our own experience using HCD methods to advance patient-centered care (PCC) in HIV treatment in Zambia.^14–17^ The literature review summarizes the following: (1) outcomes to which HCD has been applied, (2) methods used, (3) results and effectiveness, and (4) lessons learned. We present HCD-derived case study insights that could be informative to others seeking to optimize the delivery of PCC HIV interventions in transferable contexts. In addition, we hope this article will call attention to opportunities to advance HCD as a tool for adapting implementation strategies to particular contexts and end-user populations to strengthen the public health response to HIV.

## METHODS

### Literature Review

We conducted a narrative literature review^18,19^ of published articles and the grey literature on HCD in the global HIV response. Article inclusion criteria are as follows: (1) published through the search date of May 22, 2019, (2) related to HIV, and (3) presented data on HCD. Articles were excluded if they did not describe a design process or if they explicitly attributed their methods to non-HCD methodology (see Appendix 1, Supplemental Digital Content, http://links.lww.com/QAI/B389. PubMed search strategy). To identify the grey literature, we conducted targeted searches of websites from 3 HCD design leaders^6^ and another organization using HCD for health known to the authors, including any HIV-related cases on the website as of the search date. Case studies not articulating HCD methods or results were excluded. LB screened identified titles, abstracts, and case summaries using the inclusion criteria. LB and AB reviewed relevant full-text articles, abstracting the study author, participants and setting, HCD methods described, results, strengths, limitations, and lessons learned.

### Human-Centered Co-Design Workshop

We supplement the literature review with our experience applying HCD to shape a PCC intervention. The “Person Centred Public Health for HIV Treatment in Zambia Study” (PCPH) is a stepped wedge, cluster-randomized trial of a PCC intervention to improve HIV patient retention in care in Lusaka, Zambia, starting in mid-2019 implemented by the Centre for Infectious Disease Research in Zambia (CIDRZ), a partner to the Ministry of Health in HIV service provision since 2003. Our previous research in Zambia identified poor patient–provider interactions^20–22^ and a desire for differentiated care delivery options^20,23^ as drivers of poor retention, with high health facility-level variability.^24^ In response, the PCPH study's intervention was conceived to comprise (1) training of health care workers (HCW) in the principles and practices of patient-centeredness; (2) collecting and sharing data with HCW on the patient experience at the health facility; (3) HCW coaching; (4) supporting facility-level quality improvement; and (5) incentives for improved practice.

Before trial implementation, we undertook a pilot study implementing elements 1–3 of the planned intervention in 2 facilities to understand the context and test and refine the intervention. We conducted formative research on intervention components using interviews and focus-group discussions (FGDs). We then held a 5-day HCD workshop to engage HCWs who experienced the pilot in co-design activities to further refine the study intervention. Key HCD workshop strategies included the following: (1) developers cultivating empathy with HCWs and learning from insights and experiences, (2) developers and HCWs collaborating in investigation and creative problem-solving, and (3) defining actionable approaches for intervention improvement. Drawing on previous research and pilot findings, we defined 3 “How Might We…?” questions^9^ (HMW) to guide the workshop:

- Coaching: How might coaches be best positioned in health facilities to guide and support HCWs in delivering PCC according to best practices and in ways that are appropriate to facility context?;
- HCW support and motivation: How might we foster a workplace culture that empowers and motivates health care providers to provide PCC?;
- Information management: How might we make new and existing information on patient experience and patient outcomes accessible, desirable, and useable for facility staff and other key users?

The design workshop included 31 purposefully^25^ invited HCWs (users) from the pilot facilities (see Appendix 2, Supplemental Digital Content, http://links.lww.com/QAI/B389), 6 district health management team representatives (influencers) and 12 research team members (developers). The workshop agenda (see Appendix 3, Supplemental Digital Content, http://links.lww.com/QAI/B389) included common HCD insight gathering activities such as “journey maps”^9^ and personas^26^ to realize the workshop strategies. Workshop facilitation was led by an external HCD expert and co-facilitated by research team members.

To synthesize insights, participants generated visualized activity outputs common to HCD (see Appendix 4, Supplemental Digital Content, http://links.lww.com/QAI/B389). The developers took notes during each workshop session and, each day, reviewed outputs and notes, and dialogued to identify key questions, emergent insights, and direct feedback on intervention components. Developers then categorized the insights through mapping and rapid thematic analysis^8^ and proposed intervention revisions. Critical insights and themes were discussed with HCWs.

### Ethics

Study activities were conducted under a health facility-level waiver of consent, approved by the University of Zambia Biomedical Research Ethics Committee and University of Alabama at Birmingham IRB.

## RESULTS

### Literature Review

**FIGURE 1.**
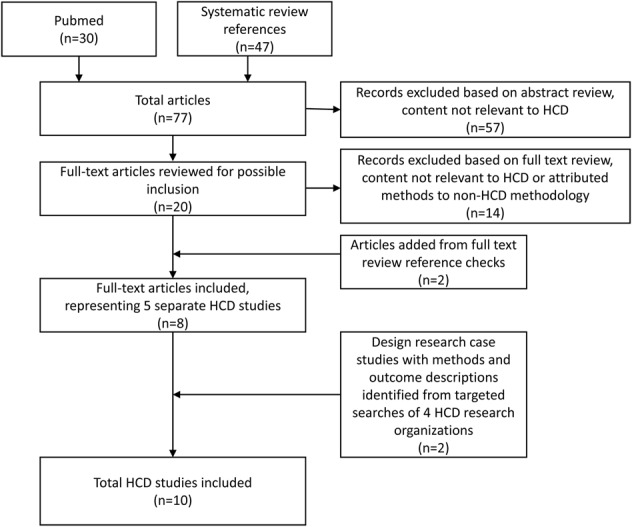
Narrative literature review HCD study inclusion.

The search strategy identified 77 published articles, of which 8 were relevant to the research question, representing 5 studies. The grey literature searching identified 4 studies, of which 2 had sufficient information for inclusion (Fig. 1). Studies came from Nigeria,^27^ Kenya,^28^ South Africa,^26,29,30^ Uganda,^26^ and the United States^31–36^ and included adults and youth. Four studies designed an mhealth tool,^27,31–36^ 1, a management process,^28^ and 2, PrEP delivery approaches with young women.^26,29,30^ All studies specified a phased process, including each of the elements described in Table 1. The order, structure, and intensity of those elements differed by the study. All but one included study^36^ articulated plans for a feasibility or effectiveness evaluation of the outcome resulting from the HCD process. However, although planned for 1 case study,^37^ no studies estimated the effect of the HCD process itself by comparing the implementation of the outcome designed to either (1) outcome implementation not informed by users or (2) informed by another formative research approach (Table 2).

**TABLE 2.**
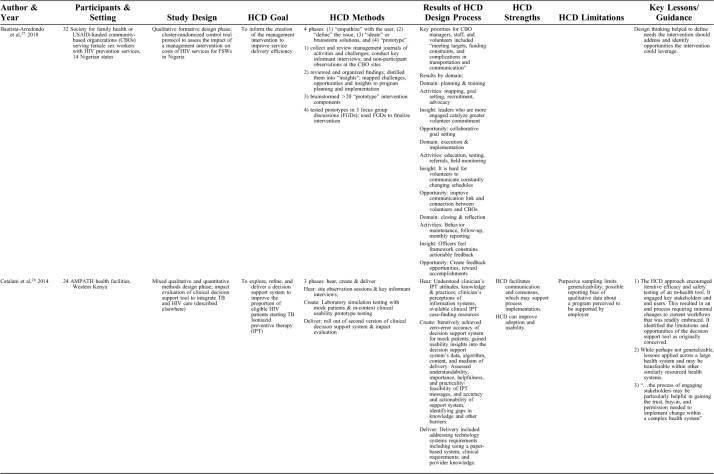

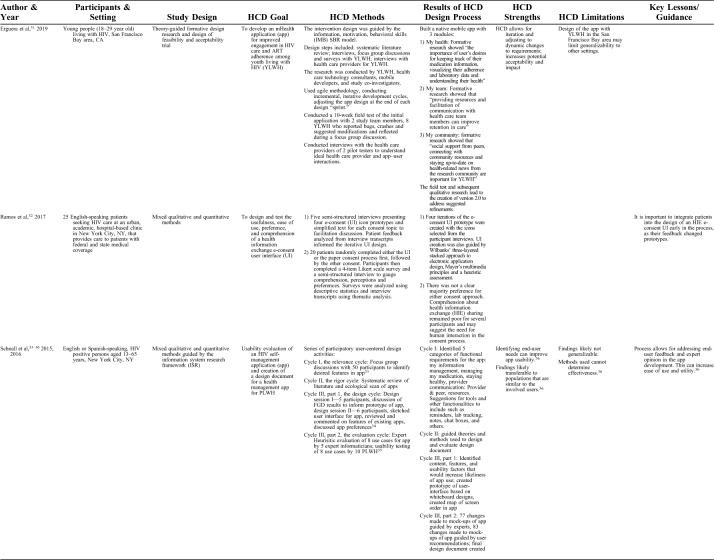

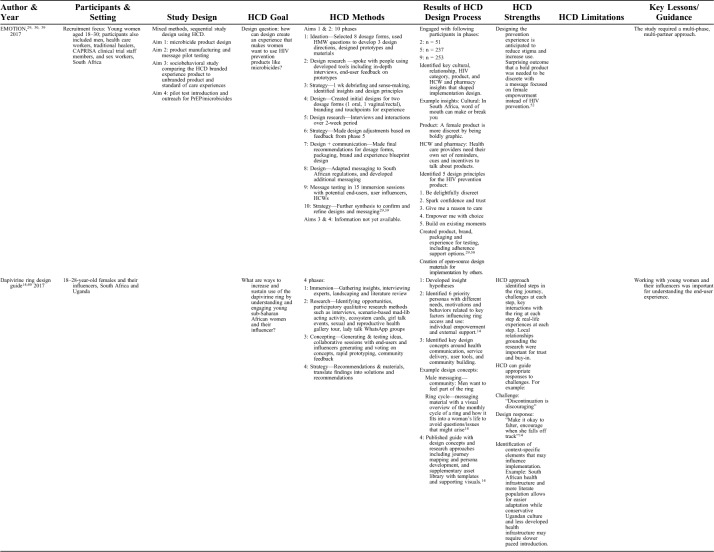
Literature Review Results on Use of HCD in HIV

In each study, the HCD application was guided by the current state of research and the user behaviors desired by the developers. Most of the studies sought to create and optimize a specific outcome (ie, a management intervention to improve efficiency,^27^ a decision support tool to integrate HIV and TB clinical care,^28^ and a mobile application to improve HIV management or patient decision-making^31,32,36^), the basic form of which was justified by previous research. Two studies,^29,38^ both focused on a newer product and a less well-understood user group (PrEP for young women), sought more broadly to understand experiences and approaches relevant to increasing and sustaining PrEP use and then proceeded to design and refine specific PrEP products and processes. Articles reflect that even when specific products are of interest, HCD processes require sufficient flexibility during the user-driven investigation stages to allow for user priorities to guide the focus and form of the final solutions designed. For example, although based on an electronic medical record system, the final HIV-TB integration support tool design utilized paper-based messaging to clinicians as the most feasible form of communication.^28^

All published studies identified beneficial changes to the product/process being designed. This represents the poten6al for HCD application. Potential publication bias may limit available data on HCD processes that failed to yield meaningful, ac6onable insights. Ramos et al,^32^ however, conclude that the HCD-improved HIE user interface still resulted in poor HIE comprehension and suggest that human interaction may be necessary for understanding. This may demonstrate that 1 cycle of iteration and testing may be insufficient to identify an optimized implementation design, indicating that implementers planning to use HCD need to allow time, resources, and/or strategies to support sufficient re-design and testing.

An HCD approach may itself be an implementation strategy, in addition to providing formative research to optimize and intervention. Catalani et al^28^ conducted their HCD work with stakeholders in the same health system where their intervention would be implemented. The authors reflect that the user engagement required for HCD facilitated the trust and acceptance required to intervene in a complex health system.

An extant literature suggests that HCD may be particularly apt for improving the usability of a specific product or tool for a relatively well-defined user group; identifying and addressing relevant concerns in a complex system or process; and identifying and avoiding implementation barriers for a new product or product access by a poorly understood user group. The limitations of HCD raised by study authors were poor generalizability of the designed outcomes^28,31,34^ and the inability of HCD as an approach to estimate effectiveness.^36^

### Human-Centered Co-Design Workshop

Our application expanded HCD to a behavioral service delivery target: changing HCW patient-centered beliefs, attitudes, and practices. Like several published studies, we aimed to refine an evidence-based intervention with predetermined components based on extensive previous and formative research. The depth of the interactions between the HCWs and research team members during the workshop, as well as the creativity encouraged by the facilitator, allowed for a breadth and openness of conversation not achieved during the formative focus group discussions. The insights gained during our workshop may be partially explained by the open relationships established and intervention feedback received during the pilot.

HCD revealed that patient-centeredness principles (eg, understanding the whole person, 2-way communication) resonated with HCW but met resistance with some HCWs such as time constraints and beliefs that punitive measures improve patient compliance. During HCD activity dialogues, HCW participants shared stories of using kindness to help struggling patients to re-engage in care, highlighting the ability to implement PCC under less-than-ideal conditions and the benefits of positive interactions. The explicit encouragement of HCD to offer multiple perspectives and dialogues and embrace a co-creative environment allowed for the interactions, which revealed these various perspectives. The key points of resonance with PCC (Table 3) and resistance (Table 4) were voiced directly or indirectly by HCWs and codified through rapid thematic analysis. While an unanticipated HCD workshop result, they present both likely challenges and promising solutions to promoting PCC practice. They would not apply to every situation; however, they offer guidance for the trial intervention coaches to anticipate and may address barriers to PCC adoption in transferable settings.

**TABLE 3.**
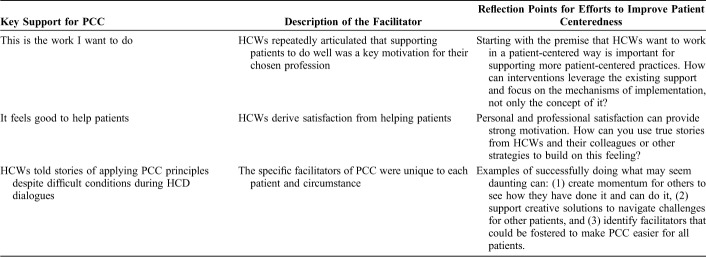
Resonance: Support Articulated for PCC by Health Care Workers

**TABLE 4.**
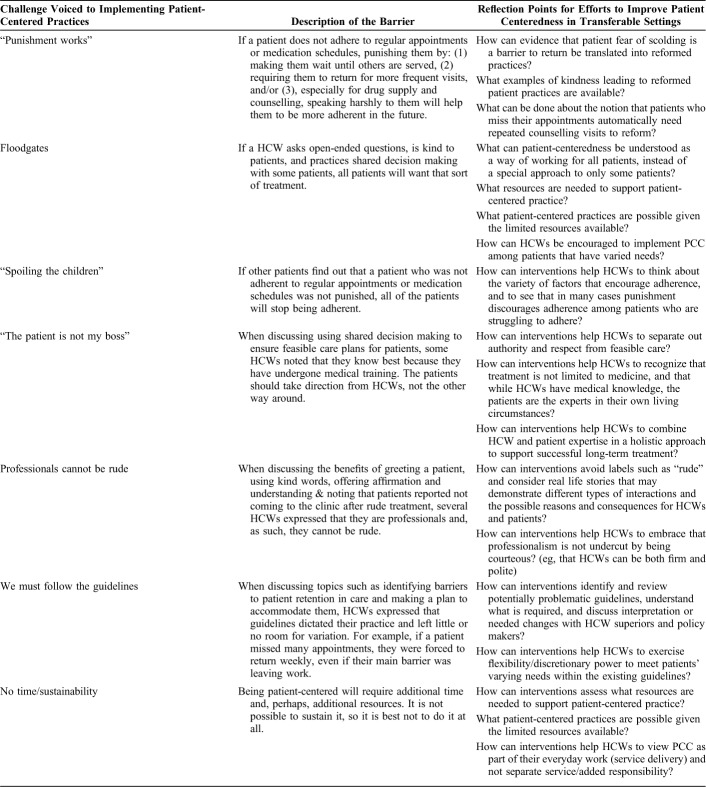

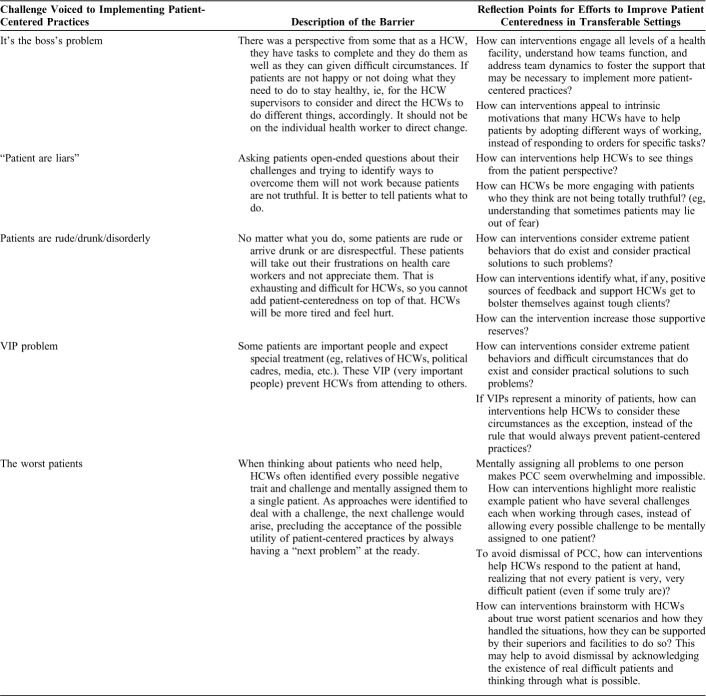

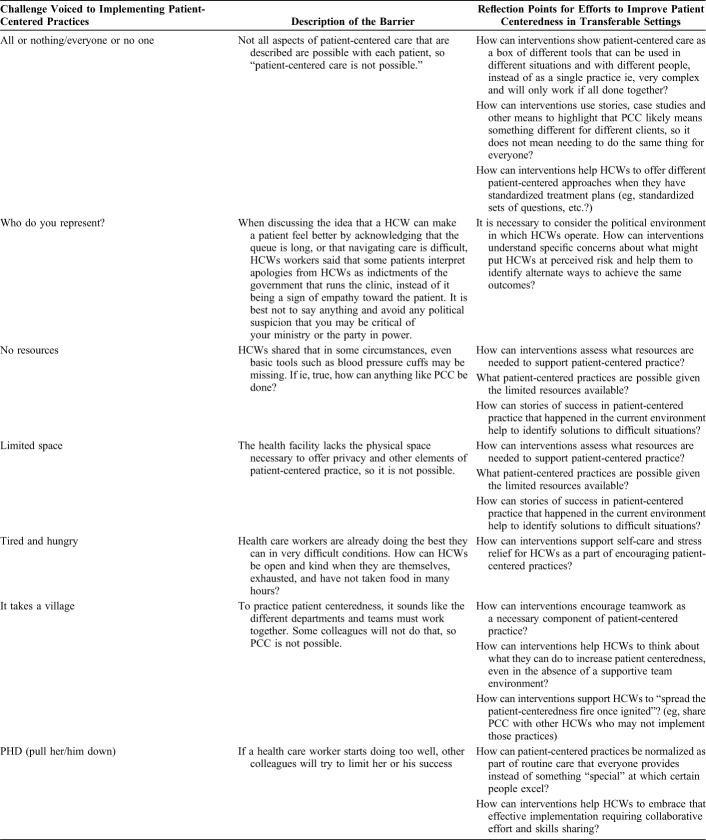
Resistance: Conceptual, Emotional, Cognitive, and Structural Challenges to Implementing Patient-Centered Practices Voiced by Health Care Workers

Intervention components that will be tested in our stepped-wedge trial were revised to improve acceptability, appropriateness, and feasibility of PCC practices, including better-using intervention mentoring and data collection to help HCWs feel visible, appreciated, and accountable. For example, HCD insights led us to augment the planned HCW FGD data collection with a quantitative HCW survey to expand and anonymize HCW feedback (additional examples in Table 5). The limited workshop days allowed for little formal iteration on proposed revisions beyond sharing ideas and eliciting additional HCW feedback. Our study, however, used further pilot period implementation to test some of the proposed changes. The rigor of our HCD process may have improved with extended time for formal iteration and testing.

**TABLE 5.**
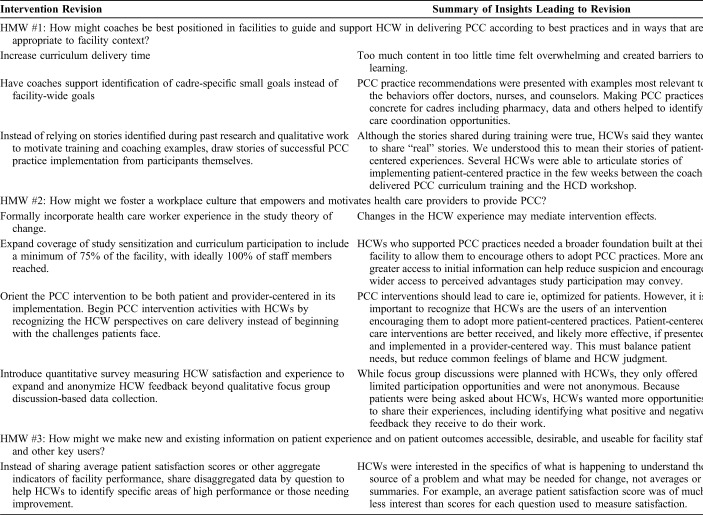
Example Revisions to PCC Intervention From HCD Workshop

## DISCUSSION

Our review suggested that HCD offers an important and emerging tool for adapting strategies to enhance ART services. While traditionally applied to products or mHealth practices, we and others have also begun to apply these principles to shape and refine the service delivery context and patient experience. Overall, HCD has the potential to improve the HIV response by more closely aligning the implementation of evidence-based products and processes with user priorities and context. In an HIV response that has to date rightly prioritized scale-up and standardization, but which now must shift to a more targeted efforts to continue improvements, HCD offers a theoretically based, robustly mapped set of practices that seek to improve outcomes through active engagement and respect for end-user views; encouragement of broad, creative inquiry; and support for iterative idea refinement in response to testing and feedback. Rigorous, successful HCD outcomes are no panacea however, and likely depend on relationships, time, and other resources required for authentic engagement and responsiveness, which may not always be available.

Extant evidence lacks an assessment of the comparative effectiveness of HCD and non-HCD processes in implementation optimization. Recent research, however, offers interesting insight into potential HCD gains. A study of the national scale-up of PrEP in Kenya identified that in addition to other stigma types, product stigma, including the similar appearance of PrEP bottles and ARV bottles, was a barrier to PrEP use.^39,40^ The HCD approach in EMOTION^29,30,37^ identified that the medical appearance of PrEP was associated with illness, creating a likely barrier to young women's uptake and use. EMOTION^29^ thus prototyped PrEP starter kits that resemble make-up bags and stickers to disguise standard labels. User engagement lessons may facilitate use in similar populations.

HCD approaches are not the only user-engagement methods that may support improved implementation. A 2017 landscape mapping of end-user research in HIV prevention for young women in sub-Saharan Africa identified 53 projects, of which 3 were explicitly HCD.^41^ There is often overlap in objectives and methods between HCD, more traditional qualitative methodologies^42,43^; participatory research^11^; engineering approaches^44,45^; and discrete choice experiments as formative research.^46^ Although efforts have been made to compare and contrast approaches,^8^ distinctions are not concrete. Authors struggled to adjudicate use of HCD in some studies in our literature review. The Schnall et al^34^ study discussed a user-centered design process but grounded their research in the Information System Research Framework and described some of their methods as participatory action research. We erred on the side of inclusion, but the multidisciplinary nature of user-centered approaches requires reviewers' interpretation of methods. HCD distinguishes itself in by focus on empathy; flexibility in inquiry as directed by users; iterative, rapid testing cycles; and emphasis on practical outcomes. Our own case study included both traditional formative research and HCD. Although our more traditional formative research raised the issue of HCW context and the need to avoid blame to increase receptivity of HCWs to PCC, the empathy established and nuance understood through the HCD workshop elevated the HCW experience to formally include improvement in the HCW experience in the study Theory of Change.

The limitations of HCD identified in the literature review including a lack of generalizability and inability to estimate effectiveness are not dissimilar to other qualitative methodologies. Instead of limitations, they are features of the approach. HCD findings may be transferable,^47^ a metric of qualitative rigor that considers the applicability of findings from one setting to a different setting with a similar context, instead of generalizable. Potential for transferability was noted in several literature review studies,^26,28,29^ as well as our own HCD case study. Critical reflection on HCD limitations would enhance future use. For example, thin preliminary knowledge of the evidence-based intervention may lead to poor design questions. An HCD process that is too rapid to allow for sufficient iteration or diversity of user engagement may produce results that lack rigor. Based on the importance of open discussion in our workshop, we believe that an absence of mutual respect and trust between users and developers could prevent empathetic interactions and produce inauthentic HCD outcomes.

An important HCD insight from our workshop was that effective implementation of an intervention to increase the patient-centeredness of care through HCW behavior change needs to be implemented in a provider-centered way. This does not diminish the central role of patients in their care processes and outcomes. It highlights that just as the lived realities of the patients' need to inform HCW engagement with patients as care partners, the lived realities of the HCWs demand that interventions to change HCW practice prioritize feasible, acceptable, and appropriate implementation strategies. HCD may be particularly appropriate in complex settings that need to consider patient, provider, and systems realities. The compendium of resonance and resistance to PCC (Tables 3 and 5) provide concrete illustrations of how using HCD can reveal challenges and opportunities that may influence intervention effectiveness. Consistent with the reflections of Catalani et al,^28^ our team felt that the communication, collaboration, and engagement between planners and users during HCD can support successful intervention implementation subsequent to the design phase.

### Limitations

Our review may have missed relevant case studies given the limited grey literature search and the practical outputs of many HCD studies, which are less likely to be published in peer-reviewed manuscripts. Similarly, we focused on HIV-related HCD, excluding possible lessons from other health areas. Owing to time and resource constraints, our HCD process did not include patients, the intended intervention beneficiaries, and key actors in their own care and treatment. Inclusion of patients would almost certainly have identified additional insights and intervention revisions. Our choice to focus on the HCWs allowed us to engage with the primary users of the planned intervention, to conduct our HCD approach in limited time, and to manage identified power dynamics within HCW cadres based on shared knowledge and vocabulary from the pilot phase.

## CONCLUSIONS

HCD has the potential to improve effective implementation of the HIV response by tailoring implementation strategies to enhance evidence-based interventions in particular service delivery settings. Although HCD is a promising and increasingly advocated ^48,49^ approach to bridge this gap; more studies that document and critically reflect on the use and impact of HCD methods in the global HIV response are needed to guide their effective use. Those seeking to promote PCC may improve implementation success by seeking out the resonance and anticipating and defusing the challenges our HCD process identified.

## Supplementary Material

SUPPLEMENTARY MATERIAL

## References

[1] UNAIDS. Miles to Go: Closing Gaps, Breaking Barriers, Righting Injustices. Geneva, Switzerland: UNAIDS; 2018.

[2] El-Sadr WM Harripersaud K Rabkin M Reaching global HIV/AIDS goals: what got us here, won't get us there. PLoS Med. 2017;14:e1002421. 29112691 10.1371/journal.pmed.1002421 PMC5675304

[3] UNAIDS. Fast-Track: Ending the AIDS Epidemic by 2030. Geneva, Switzerland: UNAIDS; 2014.

[4] Geng EH Holmes CB Research to improve differentiated HIV service delivery interventions: learning to learn as we do. PLoS Med. 2019;16:e1002809. 31112546 10.1371/journal.pmed.1002809 PMC6529020

[5] Ulett KB Willig JH Lin HY The therapeutic implications of timely linkage and early retention in HIV care. AIDS Patient Care STDS. 2009;23:41–49. 19055408 10.1089/apc.2008.0132 PMC2733237

[6] Bazzano AN Martin J Hicks E Human-centred design in global health: a scoping review of applications and contexts. PLoS One. 2017;12:e0186744. 29091935 10.1371/journal.pone.0186744 PMC5665524

[7] Giacomin J What is human centred design? Des J. 2014;17:606–623.

[8] Tolley E Traditional Socio-Behavioral Research and Human-Centered Design: Similarities, Unique Contributions and Synergies. FHI 360; 2017 Available at: heps://www.theimpt.org/documents/reports/Report-HCD-BSSResearch. pdf.

[9] Ideo.org. The Field Guide to Human-Centered Design. 1st ed IDEO; 2015 Available at: heps://www.designkit.org/resources/1.

[10] Wilson PA Valera P Martos AJ Contributions of qualitative research in informing HIV/AIDS interventions targeting black MSM in the United States. J Sex Res. 2016;53:642–654. 26241373 10.1080/00224499.2015.1016139 PMC4740277

[11] Coughlin SS Community-based participatory research studies on HIV/AIDS prevention, 2005-2014. Jacobs J Community Med. 2016;2:pii: 019. PMC5215619 28066841

[12] Teti M Koegler E Conserve DF A scoping review of photovoice research among people with HIV. J Assoc Nurses AIDS Care. 2018;29:504–527. 29576252 10.1016/j.jana.2018.02.010

[13] Rhodes SD Kelley C Siman F Using community-based participatory research (CBPR) to develop a community-level HIV prevention intervention for Latinas: a local response to a global challenge. Womens Health Issues. 2012;22:e293–e301. 22483581 10.1016/j.whi.2012.02.002 PMC3627530

[14] Beach MC Keruly J Moore RD Is the quality of the patient-provider relationship associated with better adherence and health outcomes for patients with HIV? J Gen Intern Med. 2006;21:661–665. 16808754 10.1111/j.1525-1497.2006.00399.x PMC1924639

[15] Flickinger TE Saha S Moore RD Higher quality communication and relationships are associated with improved patient engagement in HIV care. J Acquir Immune Defic Syndr. 2013;63:362–366. 23591637 10.1097/QAI.0b013e318295b86a PMC3752691

[16] Yehia BR Mody A Stewart L Impact of the outpatient clinic experience on retention in care: perspectives of HIV-infected patients and their providers. AIDS Patient Care STDS. 2015;29:365–369. 26061902 10.1089/apc.2015.0049

[17] Dang BN Westbrook RA Black WC Examining the link between patient satisfaction and adherence to HIV care: a structural equation model. PLoS One. 2013;8:e54729. 23382948 10.1371/journal.pone.0054729 PMC3559888

[18] Grant MJ Booth A A typology of reviews: an analysis of 14 review types and associated methodologies. Health Inf libraries J. 2009;26:91–108. 10.1111/j.1471-1842.2009.00848.x 19490148

[19] Greenhalgh T Robert G Macfarlane F Diffusion of innovations in service organizations: systematic review and recommendations. Milbank Q. 2004;82:581–629. 15595944 10.1111/j.0887-378X.2004.00325.x PMC2690184

[20] Zanolini A Sikombe K Sikazwe I Understanding preferences for HIV care and treatment in Zambia: evidence from a discrete choice experiment among patients who have been lost to follow-up. PLoS Med. 2018;15:e1002636. 30102693 10.1371/journal.pmed.1002636 PMC6089406

[21] Topp SM Mwamba C Sharma A Rethinking retention: mapping interactions between multiple factors that influence long-term engagement in HIV care. PLoS One. 2018;13:e0193641. 29538443 10.1371/journal.pone.0193641 PMC5851576

[22] Mwamba C Sharma A Mukamba N They care rudely!: resourcing and relational health system factors that influence retention in care for people living with HIV in Zambia. BMJ Glob Health. 2018;3:e001007. 10.1136/bmjgh-2018-001007 PMC6231098 30483408

[23] Eshun-Wilson I Mukumbwa-Mwenechanya M Kim HY Differentiated care preferences of stable patients on ART in Zambia: a discrete choice experiment. J Acquir Immune Defic Syndr. 2019;81:540–546. 31021988 10.1097/QAI.0000000000002070 PMC6625870

[24] Holmes CB Estimated mortality on HIV treatment among active patients and patients lost to follow-up in 4 provinces of Zambia: findings from a multistage sampling-based survey. PLoS Med. 2018;15:e1002489. 29329301 10.1371/journal.pmed.1002489 PMC5766235

[25] Patton MQ Qualitative Research & Evaluation Methods. 3 ed. Thousand Oaks, CA: Sage Publications, Inc.; 2002.

[26] Lin A Russell E Tyler N Dapivirine Ring Design Guide Human-Centered Design Research to Increase Uptake and Use. USAID; 2017 Available at: heps://www.usaid.gov/sites/default/files/documents/1864/V15original_new.compressed_508_fixed_final.pdf.

[27] Bautista-Arredondo S Nance N Salas-Ortiz A The role of management on costs and efficiency in HIV prevention interventions for female sex workers in Nigeria: a cluster-randomized control trial. Cost effectiveness and resource allocation. Cost Eff Resour Alloc. 2018;16:37. 30386184 10.1186/s12962-018-0107-x PMC6199740

[28] Catalani C Green E Owiti P A clinical decision support system for integrating tuberculosis and HIV care in Kenya: a human-centered design approach. PLoS One. 2014;9:e103205. 25170939 10.1371/journal.pone.0103205 PMC4149343

[29] Conrad I Launching V A Human-Centered Approach to PrEP an Implementer's Guide. Arlington, TX: Conrad.org; 2018 Available at: heps://www.conrad.org/launchingv/.

[30] Conrad V Research Summary. Available at: https://www.conrad.org/launchingv/research/. Accessed May 31, 2019.

[31] Erguera XA Johnson MO Neilands TB WYZ: a pilot study protocol for designing and developing a mobile health application for engagement in HIV care and medication adherence in youth and young adults living with HIV. BMJ Open. 2019;9:e030473. 10.1136/bmjopen-2019-030473 PMC6501960 31061063

[32] Ramos SR User-centered design, experience, and usability of an electronic consent user interface to facilitate informed decision-making in an HIV clinic. Comput Inform Nurs. 2017;35:556–564. 28481754 10.1097/CIN.0000000000000356 PMC5671918

[33] Schnall R Bakken S Rojas M mHealth technology as a persuasive tool for treatment, care and management of persons living with HIV. AIDS Behav. 2015;19(suppl 2):81–89. 10.1007/s10461-014-0984-8 PMC4497931 25572830

[34] Schnall R Rojas M Travers J Use of design science for informing the development of a mobile app for persons living with HIV. AMIA Annu Symp Proc. 2014;2014:1037–1045. 25954413 PMC4419902

[35] Schnall R Bakken S Brown W Usabilty evaluation of a prototype mobile app for health management for persons living with HIV. Stud Health Technol Inform. 2016;225:481–485. 27332247 PMC5588855

[36] Schnall R Rojas M Bakken S A user-centered model for designing consumer mobile health (mHealth) applications (apps). J Biomed Inform. 2016;60:243–251. 26903153 10.1016/j.jbi.2016.02.002 PMC4837063

[37] EMOTION—Enhancing Microbicide Uptake in High-Risk End Users. Available at: https://www.prepwatch.org/wp-content/uploads/2017/06/EMOTION_mpii_2pager_June2017.pdf. Accessed May 31, 2019.

[38] Engage HCD. The Dapivirine Ring Design Guide Human-Centered Design Research to Increase Uptake and Use. Available at: http://www.engagehcd.com/dpv-ring. Accessed May 31, 2019.

[39] Were D Atkins K Musau A Manifestations of Stigma in the Context of a National Oral Pre Exposure Prophylaxis (PrEP) Scale-up Programme in Kenya. International AIDS Society Conference on HIV Science, Mexico City, Abstract MOAD0302. Mexico; 2019.

[40] Samuel K Three Forms of PrEP Stigma in Kenya; 2019 Available at: http://www.aidsmap.com/news/jul-2019/three-forms-prep-stigma-kenya. Accessed August 12, 2019.

[41] Manager HPM End-User Research Landscape Mapping & Findings. New York, NY: AVAC; 2017.

[42] Montoya JL Georges S Poquette A Refining a personalized mHealth intervention to promote medication adherence among HIV+ methamphetamine users. AIDS Care. 2014;26:1477–1481. 24911433 10.1080/09540121.2014.924213 PMC4188758

[43] Garvie PA Lawford J Flynn PM Development of a directly observed therapy adherence intervention for adolescents with human immunodeficiency virus-1: application of focus group methodology to inform design, feasibility, and acceptability. J Adolesc Health. 2009;44:124–132. 19167660 10.1016/j.jadohealth.2008.05.006 PMC2659610

[44] Guthrie KM Rosen RK Vargas SE User input in iterative design for prevention product development: leveraging interdisciplinary methods to optimize effectiveness. Drug Deliv Transl Res. 2017;7:761–770. 28653286 10.1007/s13346-017-0397-0 PMC6103777

[45] Guthrie KM Rohan L Rosen RK Vaginal film for prevention of HIV: using visual and tactile evaluations among potential users to inform product design. Pharm Dev Technol. 2018;23:311–314. 28592183 10.1080/10837450.2017.1339085 PMC5740013

[46] Terris-Prestholt F Neke N Grund JM Using discrete choice experiments to inform the design of complex interventions. Trials. 2019;20:157. 30832718 10.1186/s13063-019-3186-x PMC6399844

[47] Tobin GA Begley CM Methodological rigour within a qualitative framework. J Adv Nurs. 2004;48:388–396. 15500533 10.1111/j.1365-2648.2004.03207.x

[48] Lin AH Breger TL Barnhart M Learning from the private sector: towards a keener understanding of the end-user for microbicide introduction planning. J Int AIDS Soc. 2014;17(3 suppl 2):19162. 25224619 10.7448/IAS.17.3.19162 PMC4163992

[49] Cheney C Gates Foundation and USAID Team up to Bring Design to Health: Inside Development Global Health; 2018 Available at: https://www.devex.com/news/gates-foundation-and-usaid-team-up-to-bring-design-to-health-92084. Accessed May 31, 2019.

[50] IDEO. Empowering Women to Prevent HIV. 2019 Available at: heps://www.ideo.com/case-study/empowering-women-to-prevent-hiv.

