# Supplementary material for: Human-Centered Design Lessons for Implementation Science: Improving the Implementation of a Patient-Centered Care Intervention
Source: J Acquir Immune Defic Syndr. 2019 Nov 26;82(3):S230–43. doi: 10.1097/QAI.0000000000002216 (PMC6880397; doi:10.1097/QAI.0000000000002216)

Supplemental Appendix 1:

PubMed Search Strategy

We searched PubMed using the following terms: (HIV) AND ("human centered design" OR "human centred design" OR "user centered design" OR "user centred design" OR "design thinking" OR "human centered design approach" OR "human centred design approach" OR "human centered design principles" OR "human centred design principles" OR "human centered design process" OR "human centred design process"). Additionally, we conducted secondary reference review on all articles included in the review and two systematic reviews identified in the search^53,54^.

Supplemental Appendix 2:

Health Care Worker Cadres Participating in Human Centered Co-Design Workshop

1. In-charges: Facility, Outpatient Department and ART Department
2. Clinical Officer
3. Nurse
4. Midwife
5. Lay health care worker
6. Pharmacy
7. Data
8. Laboratory Technologist
9. Clerk
10. Clinical Care Specialist
11. Health Information Officer
12. Psychosocial counsellor
13. District Nursing Officer
14. District Planner

Supplemental Appendix 3:

Agenda / List of human-centered co-design activities utilized during workshop

- Patient Empathy Mapping
  - Patient Journey Maps
  - Patient Personas
- Small group dialogues on patient-centered care in practice
- Body-mapping the information eco-system in health facilities
- ‘Data Dashboard’ mockup, providing feedback on prototype of data sharing mechanism
- Coach / Healthcare Worker journey mapping
- Healthcare worker and District Management Office staff member dialogues and knowledge sharing
- Sharing PCPH study formative research findings on health care worker motivation
- Group dialogue about reward mechanism / incentive options
- “The Great Egg Drop” – creative play and team building activity
- Improvisational energizers
- Vision Posters and Storyboarding: “Tomorrow’s Headline” for where implementing more patient-centered care will get you as a cadre in 1 year
- Prototyping and revision of Healthcare Worker Survey Questionnaire
- Storytelling

Supplemental Appendix 4:

Example visualized HCD activity outputs

1. Patient Journey Map


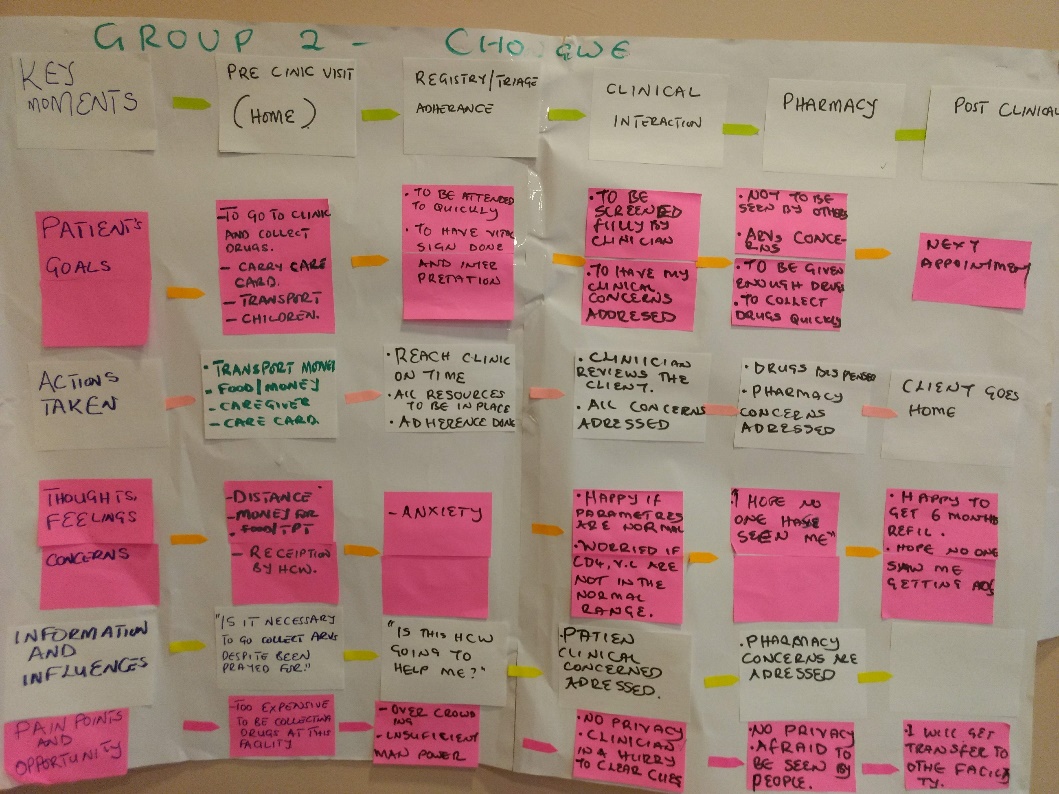


1. Vision Posters and Storyboarding: “Tomorrow’s Headline” for where implementing more patient-centered care will get you as a cadre in 1 year


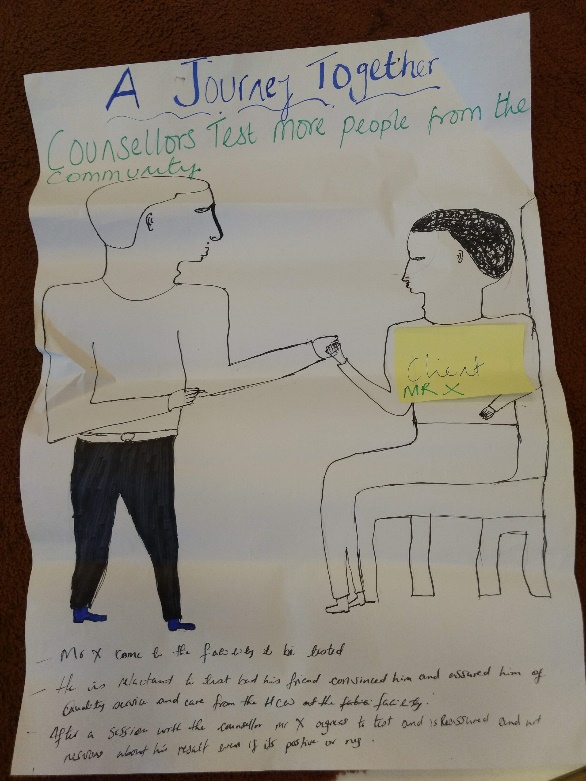


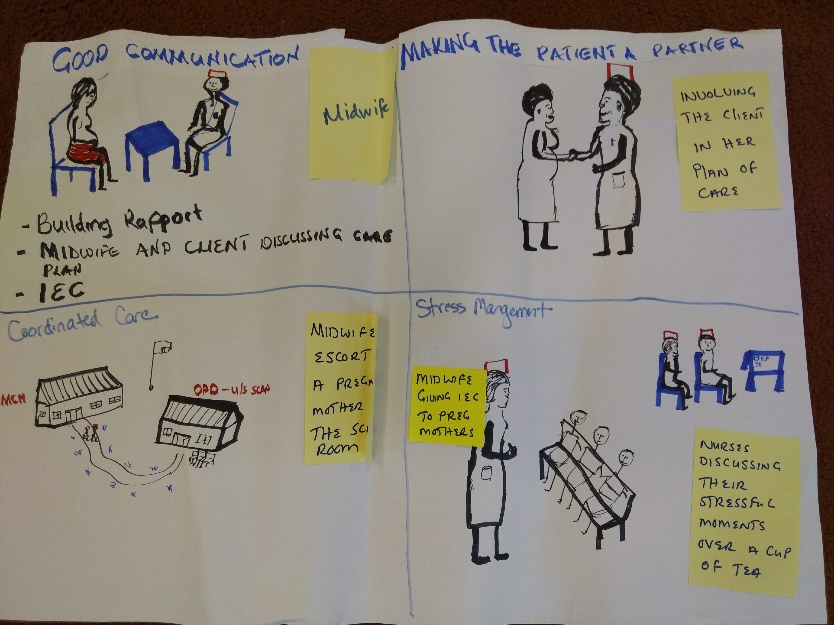


1. Coach/ HCW Journey Mapping of Pilot Intervention Components


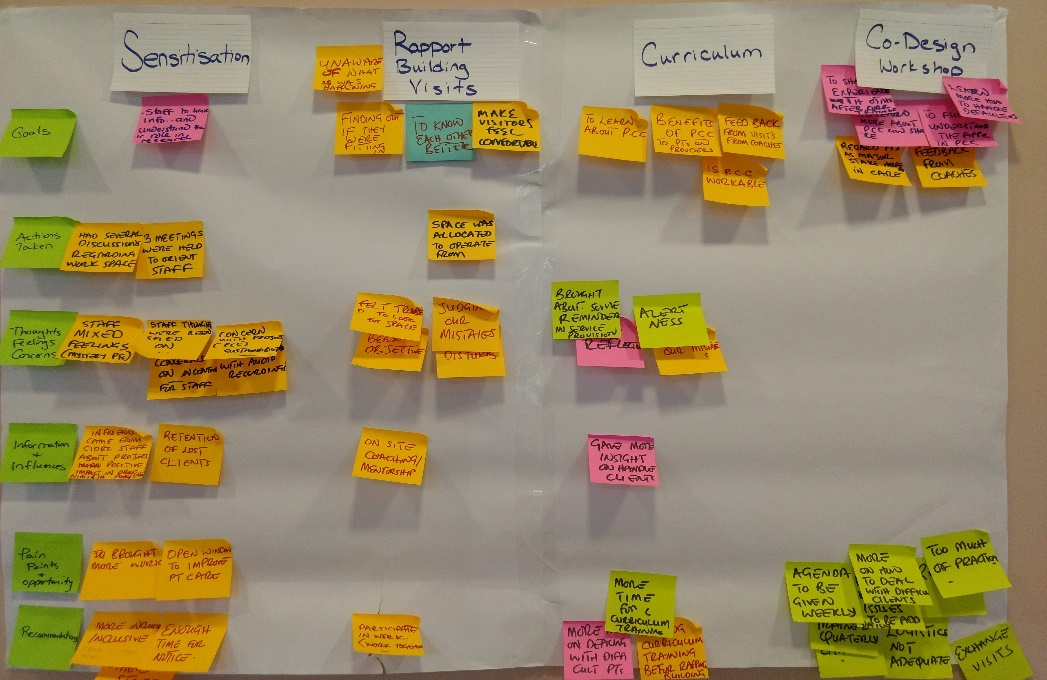

Supplement: SUPPLEMENTARY MATERIAL [file qai-82-s230-s001.docx]
